# Professor José Miguel Barea (1942–2018): a tribute to an inspiring scientist

**DOI:** 10.1007/s00572-018-0837-9

**Published:** 2018-06-07

**Authors:** Peter Jeffries

**Affiliations:** School of Biosciences, University of Kent, Canterbury, Kent CT2 7NJ UK

**Keywords:** Arbuscular mycorrhizas, Mycorrhiza conferences, Teacher, Colleague, Remembrances

## Abstract

The mycorrhiza and, more generally, soil microbiology research communities recently have lost one of their most ardent scientists. José Miguel Barea was a world leader of arbuscular mycorrhiza research and pioneered the establishment of such studies in Spain and Latin American. He was a prolific publisher, enthusiastic teacher of many graduate students and a genial host to visitors of his beloved Granada. He will be missed wherever mycorrhizasts gather.

José Miguel Barea (Fig. 1) died in hospital on the 3rd of April 2018 after a short illness. Despite taking formal retirement a few years ago, José Miguel was still working enthusiastically at his Institute [Estación Experimental del Zaidin (EEZ) del CSIC, Granada, Spain] as an Honorary Professor until illness curtailed his activities. He will be remembered not only for his prolific contributions to the scientific knowledge of symbiotic microorganisms and their interactions with plants, but also for his leadership and establishment of Spanish mycorrhizal biology. He was renowned for his generous and warm hospitality and social organisational talents. A visit to Granada, hosted by José Miguel was more than just a scientific experience, and his pride in his city and culture was always very evident (and almost equal to his love of Real Madrid!).

**Fig. 1:**
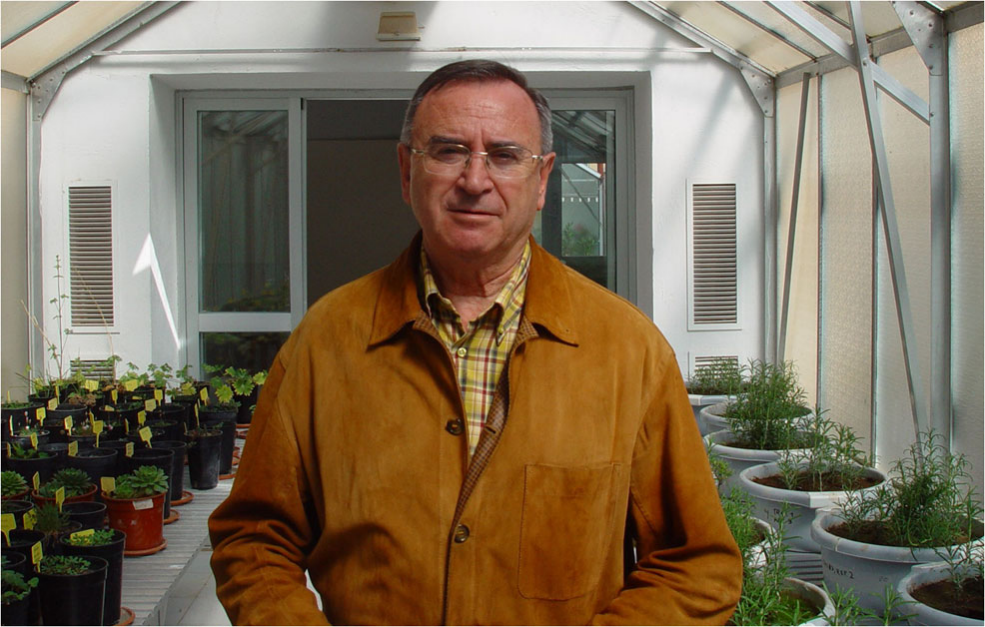
José Miguel Barea

José Miguel was a native Granadino who first graduated in Pharmacy from the University of Granada in 1965. His long association with EEZ then began as he undertook a Doctorate in the Soil Microbiology Department where he was based for the rest of his professional career, starting as a basic scientist in 1972 and rising to become Director of the Institute from 1989 to 1998. For almost 50 years, his leadership ensured that EEZ would become the first Spanish institute to study arbuscular mycorrhizas in detail and would go on to develop an international reputation for the breadth and quality of the investigations carried out therein. The Institute became a well-known centre for training of graduate students in root symbioses and a magnet for visiting researchers from around the globe. José Miguel was also keen to promote Granada as a host for International conferences and his efforts ensured that the 3rd European Symposium on Mycorrhiza (1994) and the 5th International Conference on Mycorrhiza (2006) were well attended and unforgettable scientific and social events.

José Miguel’s scientific career started with the study of soil bacteria able to solubilise sparingly available phosphate and/or producing plant hormones to improve plant nutrition and growth. His interests extended to mycorrhizal biology with a study visit to Rothamsted Experimental Station in the United Kingdom in autumn 1972, accompanied by his scientist wife Rosario (‘Charo’) Azcón. At the time, Rothamsted was a hotspot for the rapidly emerging science of arbuscular mycorrhizal biology. There he worked with Barbara Mosse, Margaret Brown and David Hayman (Fig. 2), and the first of his numerous publications on this group of fungi was published from their fruitful collaboration. Co-incidentally, Rothamsted was also where he met Silvio Gianinazzi and Vivienne Gianinazzi–Pearson, and where they forged a life-long friendship, with close ties both professionally and privately. On returning to Granada, the work in EEZ on root symbiotic systems rapidly expanded and a Nature (Azcón-Aguilar et al. 1979) paper, co-written with Charo and her sister Conchi Azcón-Aguilar, was a key result. Over the subsequent decades, the research groups of all three family members expanded as EEZ became a European centre for mycorrhiza research encompassing all aspects of the biology, physiology, technology and application of these fascinating fungi. More recently, the Institute has gained an excellent reputation for studies of molecular biology of AMF and their role in ecosystem restoration, encouraged again by the foresight of José Miguel. The application of mycorrhizal fungi to sustainable crop production and their interactions with growth-promoting rhizobacteria was always strongly featured in José Miguel’s research, and he developed good relations with the biotechnology industry, farmers and growers within the local region.

**Fig. 2:**
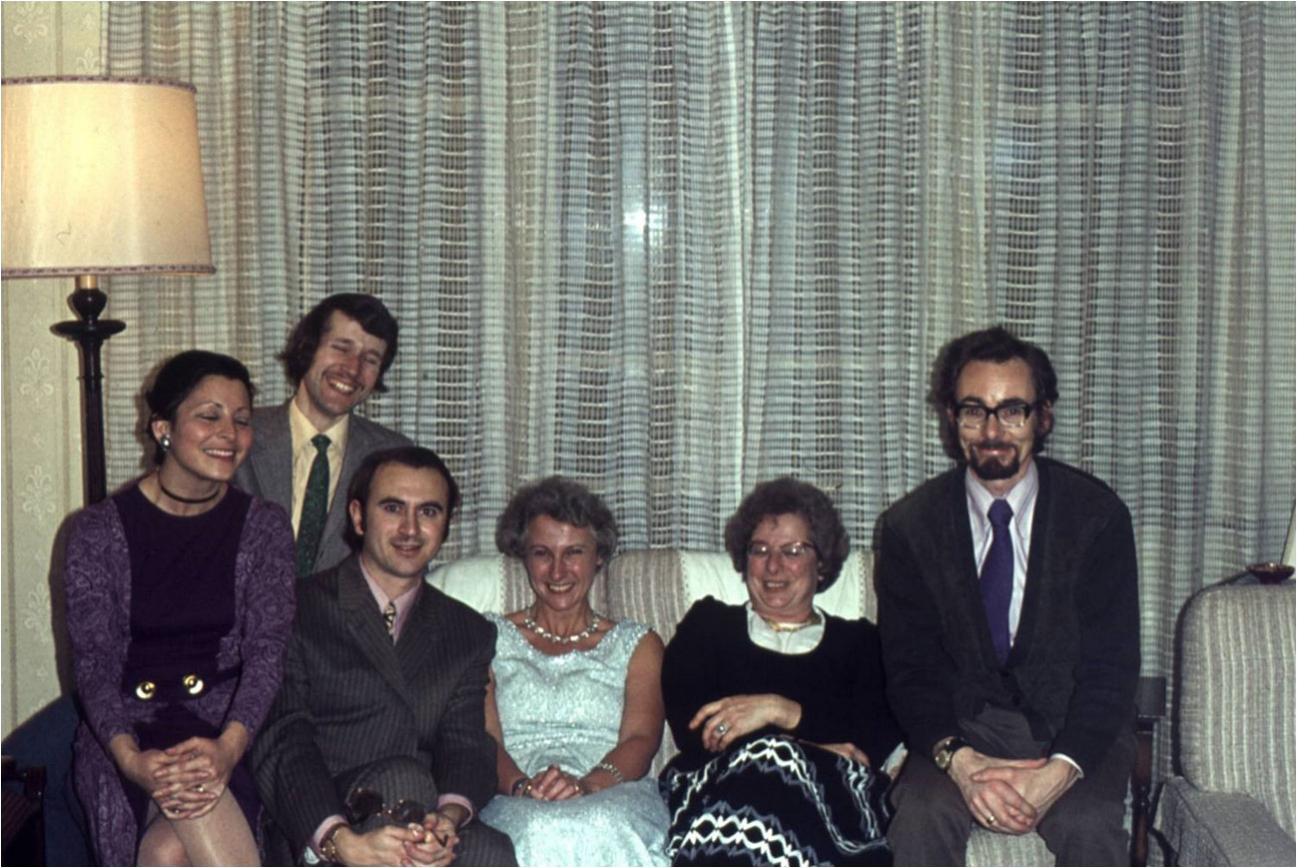
At Rothamsted, 1972. Front row from left: Rosario Azcón, José Miguel Barea, Margaret Brown, Barbara Mosse, David Hayman; (back row: name not available)

In terms of his personal legacy, José Miguel published over 170 papers in good quality SCI-refereed journals, wrote many chapters in international and national books and authored numerous technical articles and contributions to congress proceedings. He was a workaholic and was often at his desk at seven o’clock in the morning, weekends included. He was not only an inspiring scientist but also an enthusiastic teacher. Over 30 students undertook doctoral theses under his direction, and several of these young researchers have become leading names in their research field. He taught in many graduate courses in Granada, Murcia, the Magreb and in Latin America, an area where he was especially keen to promote the development of mycorrhiza research. He was particularly fond of Chile and frequently visited his colleagues at the University de la Frontera, Temuco. Fernando Borie writes: ‘I knew Jose Miguel at the beginning of 1977 when I started my Doctoral Program at Granada University under his guidance. During that time I was joined by many other Latin American students, from Venezuela, Cuba, Brazil and México for example, which proved a key element in disseminating mycorrhizal knowledge across our Continent. We will never forget the versatile and friendly José Miguel. It was a gift for us to share with him a myriad of enjoyable moments.’

Besides his International work, José Miguel directed many Spanish National Projects and was leader or partner in several EU research programmes. His links with other European researchers were strong, and he was Vice-Chairman of a number of very successful EU-COST Actions on arbuscular mycorrhiza from 1995 to 2005. As mentioned earlier, he and his team were excellent organisers and hosts at several European and International conferences that were held in Granada, and his influence on the social elements of the events always was very evident. He acted as consultant for numerous bodies including FAO and IAEA, and, as his career progressed, he received numerous accolades and honorary appointments to other Academies and Institutions such as the Universities of Granada, Buenos Aires and Temuco.

On a personal level, I first met José Miguel on a brief invited visit after I had contacted him about partnering with him for a joint grant application to the European Commission. I remember our first encounter well – a warm welcome at Granada airport and a short car journey into the city where our first stop was for ‘a quick beer’ below the lights of the magnificent Alhambra, followed by a rapid night-time tour of the Albaicin and a ‘nightcap’ before eventually delivering me to my hotel. I soon learnt that this was nothing atypical but reflected José Miguel’s philosophy of a life to be enjoyed and not taken too seriously outside his long working hours. In social interactions, he always was charming and a ‘gentleman’ in the old-fashioned sense of the word. Although that first grant application was not successful, it lead to my spending several months on sabbatical working in the greenhouses at EEZ and putting together a successfully funded project working on the role of symbiotic microorganisms in the rehabilitation of desertified ecosystems in Almeria. This allowed me to make further visits to Granada to integrate into the close-knit community of the EEZ soil microbiologists and to share many of his friends both within and outside the Institute. I also got to appreciate the frequent choral performances of the local singing group of which José Miguel was a valued member – he often would burst into song whilst driving us (as he usually did) to the various field trips we engaged in during the project work. We collaborated on a number of publications and reviews where he always insisted he would leave the factual material alone but would ‘correct my English’.

Words that come together from people who have known José Miguel recall him as a wonderful, friendly person and an excellent, inspiring scientist who has left a rich legacy of knowledge to future mycorrhizasts. He was proud of his family, and will be sadly missed by Charo and his four grown-up children, Patricia, José Miguel, Ignacio and Luís, and his four grandchildren.
